# The use of multiple imputation (MI) in cluster randomised trials with suspected missing not at random (MNAR) outcome

**DOI:** 10.1186/1745-6215-16-S2-P143

**Published:** 2015-11-16

**Authors:** Rebecca Playle, Elinor Coulman, Dunla Gallagher, Sharon Simpson

**Affiliations:** 1 South East Wales Trials Unit, Cardiff University, Cardiff, UK; 2 University of Glasgow, Glasgow, UK

## Introduction

The HELP trial of a healthy lifestyle and eating programme for obese pregnant women resulted in a final response rate of 70%. More women in the intervention arm than the control arm were lost to follow-up, leading the trial team to suspect a MNAR mechanism for missing outcome data.

## Methods

Missing-ness in the primary outcome (BMI) was explored in relation to all baseline demographic and post-randomisation variables using logistic regression. An imputation model was developed with cluster (study site) included as a factor together with significant predictors of missing outcome, variables in the primary analysis model, variables used to balance the randomisation and the outcome. The number of imputations performed was equal to the proportion of missing data. Sensitivity analyses using two approaches were performed on the pooled primary analysis to examine the change in effect for plausible departures from missing at random (MAR) towards MNAR. MI was carried out in STATA v13.1, REALCOM-IMPUTE and SPSS v20 for comparison.

## Results

Opinions were gathered from the study team to gauge the extent to which they thought BMI data would differ in those who dropped out prior to revealing the results of the trial. The team found this difficult to estimate in order to facilitate the pattern mixture approach to sensitivity analysis. The re-weighting approach to sensitivity analysis was a suitable alternative in this study.

## Discussion

The pitfalls, challenges and practical considerations of using MI and sensitivity analyses in cluster trials with MAR or MNAR will be discussed.

